# In Vivo Investigation of the Ameliorating Effect of Tempol against MIA-Induced Knee Osteoarthritis in Rats: Involvement of TGF-β1/SMAD3/NOX4 Cue

**DOI:** 10.3390/molecules26226993

**Published:** 2021-11-19

**Authors:** Hagar B. Abo-zalam, Rania M. Abdelsalam, Rehab F. Abdel-Rahman, Mohamed F. Abd-Ellah, Mahmoud M. Khattab

**Affiliations:** 1 Department of Pharmacology and Toxicology, Faculty of Pharmacy, October 6 University, Giza 12585, Egypt; 2 Department of Pharmacology and Toxicology, Faculty of Pharmacy, Cairo University, Cairo 11562, Egypt; rania.mohsen@pharma.cu.edu.eg (R.M.A.); mahmoud.khattab60@gmail.com (M.M.K.); 3 Department of Biology, Faculty of Pharmacy, New Giza University, Cairo 12613, Egypt; 4 Department of Pharmacology, Medical Research and Clinical Studies Institute, National Research Centre, Giza 12622, Egypt; rehabs2001@yahoo.com; 5 Department of Pharmacology and Toxicology, Faculty of Pharmacy, Al-Azhar University, Cairo 11651, Egypt; mohamedabd_ellah@yahoo.com

**Keywords:** osteoarthritis, monosodium iodoacetate, tempol, NOX4

## Abstract

Osteoarthritis (OA) is a complex disease characterized by structural, functional, and metabolic deteriorations of the whole joint and periarticular tissues. In the current study, we aimed to investigate the possible effects of tempol on knee OA induced by the chemical chondrotoxic monosodium iodoacetate (MIA) which closely mimics both the pain and structural changes associated with human OA. Rats were administrated oral tempol (100 mg/kg) one week post-MIA injection (3 mg/50 μL saline) at the right knee joints for 21 consecutive days. Tempol improved motor performance and debilitated the MIA-related radiological and histological alterations. Moreover, it subsided the knee joint swelling. Tempol decreased the cartilage degradation-related biomarkers as matrix metalloproteinase-13, bone alkaline phosphatase (bone ALP), and fibulin-3. The superoxide dismutase mimetic effect of tempol was accompanied by decreased NADPH oxidase 4 (NOX4), inflammatory mediators, nuclear factor-kappa B (NF-κB), over-released transforming growth factor-β1 (TGF-β1). Tempol decreased the expression of chemokine (C-C motif) ligand 2 (CCL2). On the molecular level, tempol reduced the phosphorylated protein levels of p38 mitogen-activated protein kinase (MAPK), and small mother against decapentaplegic 3 homologs (SMAD3). These findings suggest the promising role of tempol in ameliorating MIA-induced knee OA in rats via collateral suppression of the catabolic signaling cascades including TGF-β1/SMAD3/NOX4, and NOX4/p38MAPK/NF-κB and therefore modulation of oxidative stress, catabolic inflammatory cascades, chondrocyte metabolic homeostasis.

## 1. Introduction

Osteoarthritis (OA) is a chronic degenerative disease that causes articular cartilage loss, subchondral bone sclerosis, and osteophyte formation. OA affects the knees, hips, hands, and spine [1,2]. Chronic pain, joint instability, stiffness, and radiographic alterations are the main clinical symptoms [3].

The intra-articular injection of monosodium iodoacetate (MIA) is considered a convenient model for knee OA [4]. MIA is a simple, non-invasive, and fast-progressing chemically induced animal model of OA which is mainly used to evaluate pain behavior [5,6]. MIA directly affects the metabolic balance activity of chondrocytes by disrupting glycolysis as a result of inhibiting the activity of the glyceraldehyde-3-phosphate dehydrogenase (GAPDH) enzyme, which eventually leads to cartilage degeneration [7]. MIA exhibits biphasic pain behavior; the early phase is the inflammatory phase (2–6 days post-injection) and the pain is the late phase (>14 days post-injection) [8]. MIA induces chondrocyte death with early human OA-like functional, radiological, histological, and biochemical alterations [9,10,11].

The abnormal production of cytokines, chemokines, reactive oxygen species (ROS), and matrix metalloproteinases (MMPs) by chondrocytes or synoviocytes has been linked to OA pathogenesis which in consequence triggers degradation of extracellular matrix (ECM) components and dysregulates chondrocyte metabolism leading to an imbalance between cartilage matrix degradation and synthesis [12]. Hence, chondrocytes undergo metabolic surplus of dual up-regulation of catabolic and anabolic factors secretions occurs as a compensatory repair attempt via downstream signal transduction pathways [13,14]. Consequently, the vicious stress circles of oxidative stress and inflammation are continued leading to cartilage damage [15]. A variety of MMPs are produced by OA chondrocytes, among which is the major catabolic effector in OA, MMP-13, which contributes mostly to degrade collagen type II as well as proteoglycans and other ECM components [16].

In early OA, chondrocytes stimulate transforming growth factor-β1 (TGF β) to directly activate the canonical signal transducer small mother against decapentaplegic 3 homologs (SMAD3) to repair cartilage degeneration [17], which subsequently activates the target transcription of NADPH oxidase 4 (NOX4) [18]. The overexpression of NOX4 leads to excessive ROS production (especially H_2_O_2_), which activates the downstream p38 mitogen-activated protein kinase (P38MAPK) phosphorylation causing deleterious signaling events [19,20].

In January 2020, the American College of Rheumatology (ACR) and the Arthritis Foundation (AF) have been updated the treatment guidelines of knee OA. Exercise, weight loss, oral, and topical NSAIDs and intra-articular corticosteroids are strongly recommended treatment options. Additionally, duloxetine, cognitive behavioral therapy, and topical capsaicin are conditionally recommended treatments. Moreover, the guidelines recommended against the use of glucosamine and chondroitin, hyaluronic acid injections, bisphosphonates, hydroxychloroquine, methotrexate, platelet-rich plasma injections, stem cell injections, tumor necrosis factor (TNF) inhibitors, and interleukin-1 receptor antagonists [21].

Tempol (4-Hydroxy-2,2,6,6-tetramethylpiperidine-1-oxyl) is a membrane-permeable radical scavenger that possesses an anti-oxidant efficacy which is emphasized through its ability to facilitate the metabolism of a wide range of ROS and reactive nitrogen species (RNS). Tempol has superoxide dismutase (SOD)-mimetic and catalase-like actions. Tempol also improves nitric oxide (NO) bioavailability and catalytically removes the highly reactive peroxynitrite (ONOOˉ) species [22]. Currently, tempol is subjected to many clinical trials sponsored by Matrix Biomed, Inc. (Los Angeles, CA, USA) such as a phase II clinical trial of treatment of radiation and cisplatin-induced toxicities with tempol, and a clinical trial on a single-patient compassionate use of tempol for the treatment of prostate cancer.

Since the pharmacologic treatments for OA have several limitations, researchers have been motivated to find alternative therapeutic agents. To our knowledge, this is the first study to focus on the In vivo therapeutic effects and mechanisms of tempol in a rat knee OA model. In 2000, Cuzzocrea et al. [23] demonstrated the beneficial effects of tempol on collagen-induced arthritis rodent model as an intracellular radical scavenger which may be useful in the treatment of associated local or systemic inflammation conditions in a collagen-induced arthritis rodent model.

Based on these considerations, the present experimental study aims to explicate the therapeutic outcomes of tempol on the different pathological aspects of MIA-induced knee OA, including; (a) functional evaluation of motor balance and pain, (b) structural alterations (using physical assessment of knee joint edema, X-ray imaging, and histopathology), and (c) knee joint degradation-related. In addition, we aimed to investigate the potential mechanisms and signaling pathways of tempol in ameliorating MIA-induced OA in rats involving; (a) oxidative phosphorylation status, (b) oxidative stress biomarkers, (c) OA-released TGF-β1, (d) intracellular signaling proteins (SMAD3, and p38MAPK), and, (e) inflammatory and pain mediators.

## 2. Results

### 2.1. Effect of Tempol on Knee Joint Swelling

**Figure 1:**
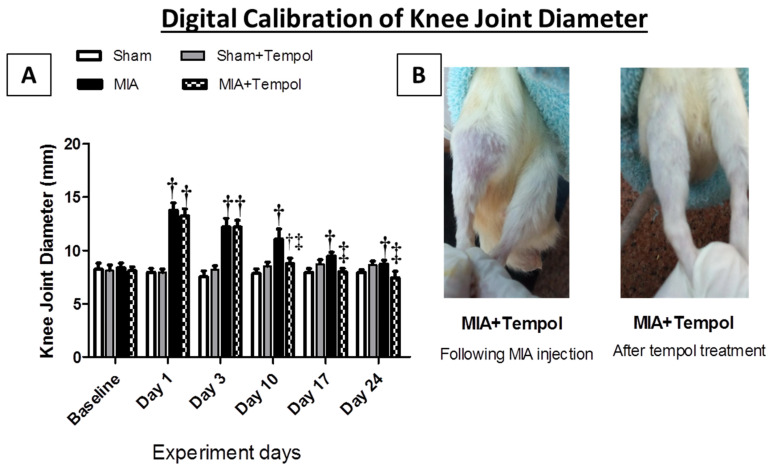
Effect of Tempol on the digital caliper test. Rats were subjected to a single intra-articular injection of 3 mg MIA/50 μL saline in their right knees, and then tempol was administered starting from the 7th day of the experiment in a dose of (100 mg/kg/day) by oral gavage for 21 consecutive days. (**A**) Values of knee joint diameter are expressed as mean ± SD (*n* = 8). Statistical analysis was carried out using repeated measures ANOVA test for factors time and group, followed by the Bonferroni test. ^†^ *p* < 0.05 vs. the sham group, and ^‡^ *p* < 0.05 vs. MIA group. (**B**) Gross image of MIA + Tempol group just after MIA injection and in the end of the experiment.

Figure 1 demonstrated the physical assessment of the knee joint edema by the digital caliper. Symptoms of the joint swelling were observed after the intra-articular injection of MIA especially at the first 10 days of the experiment schedule, where the diameter of the diseased right knee joints showed marked increases on day 1 (73.05%, *p* < 0.001), day 3 (62.04%, *p* < 0.001), and day 10 (41.38%, *p* < 0.001) as compared to the sham group. In contrast, relief in joint swelling by (20.88%, *p* < 0.001) was observed in the MIA + Tempol group starting from day 10 of the experiment (the 4th day of treatment) compared to the MIA group. On the other hand, tempol post-treated rats exhibited normal knee diameter measures at days 17 and 24, relative to sham groups.

### 2.2. Effect of Tempol on Rotarod Performance Test

**Figure 2:**
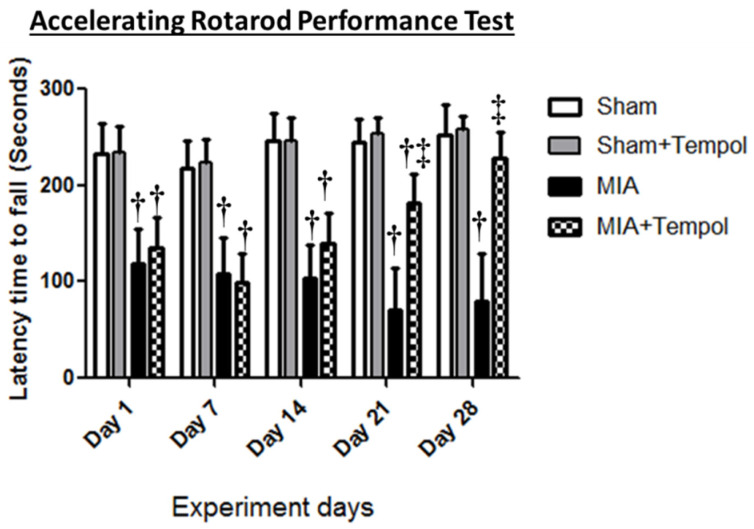
Effect of Tempol on the Rotarod test. Rats were subjected to a single intra-articular injection of 3 mg MIA/50 μL saline in their right knees, and then tempol was administered starting from the 7th day of the experiment in a dose of (100 mg/kg/day) by oral gavage for 21 consecutive days. Values of latency time to fall are expressed as mean ± SD (*n* = 8). Statistical analysis was carried out using repeated measures ANOVA test for factors time and group, followed by the Bonferroni test. ^†^ *p* < 0.05 vs. the sham group, and ^‡^ *p* < 0.05 vs. MIA group.

The accelerating rotarod was used for the functional assessment of motor balance and pain as presented in (Figure 2). Progressive motor dysfunction was observed after MIA insult, where MIA rats displayed considerable reductions in the latency time to fall at the corresponding day 1 (1.98 folds, *p* < 0.001), day 7 (2.02 folds, *p* < 0.001), day 14 (2.36 folds, *p* < 0.001), day 21 (3.43 folds, *p* < 0.001), and day 28 (3.16 folds, *p* < 0.001) when compared to the sham group. Within two weeks of MIA injection (Day 7 and 14), tempol-treated rats exhibited similar findings comparable to MIA group. On the contrary, tempol post-treatment revealed pronounced improvements in the motor coordination through significant increases of latency time to fall at day 21 (2.55 folds, *p* < 0.001), and day 28 (2.86 folds, *p* < 0.001) as compared to the MIA group.

### 2.3. Effect of Tempol on Knee Joint Degradation-Related Biomarkers

**Figure 3:**
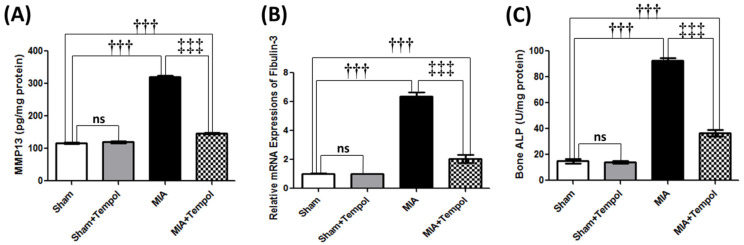
Effect of Tempol on knee joint degradation-related biomarkers on osteoarthiritic rats. Rats were subjected to a single intra-articular injection of 3 mg MIA/50 μL saline in their right knees, and then tempol was administered starting from the 7th day of the experiment in a dose of (100 mg/kg/day) by oral gavage for 21 consecutive days. Values of (**A**) Tissue contents of metalloproteinase-13 (MMP-13), (**B**) The mRNA expression of fibulin-3, and (**C**) Tissue contents of bone alkaline phosphatase (ALP). Statistical analysis was carried out using one-way ANOVA followed by Tukey’s multiple comparison test. Results are expressed as mean ± SD (*n* = 6). ^†††^ *p* < 0.001 vs. the sham group, and ^‡‡‡^ *p* < 0.001 vs. MIA group.

MIA injection resulted in extracellular matrix (ECM) degradation associated with over-release of MMP-13 contents by 2.7 folds (*p* < 0.001) as compared to the sham group (Figure 3A). Expectedly, MIA further increased mRNA expression of fibulin-3 (6.2 folds, *p* < 0.001) and bone ALP activity (6.2 folds, *p* < 0.001) when compared to the sham group (Figure 3B,C) which sensibly connected to articular damage indication. Conversely, post-treatment with Tempol reversed these elevations by (2.2 folds, *p* < 0.001) MMP-13, (3.1 folds, *p* < 0.001) fibulin-3, and (2.5 folds, *p* < 0.001) bone ALP, compared to the MIA group (Figure 3A–C).

### 2.4. Effect of Tempol on Mitochondrial Complex IV Oxidase, Oxidative Stress Biomarkers, and TGF-β1

**Figure 4:**
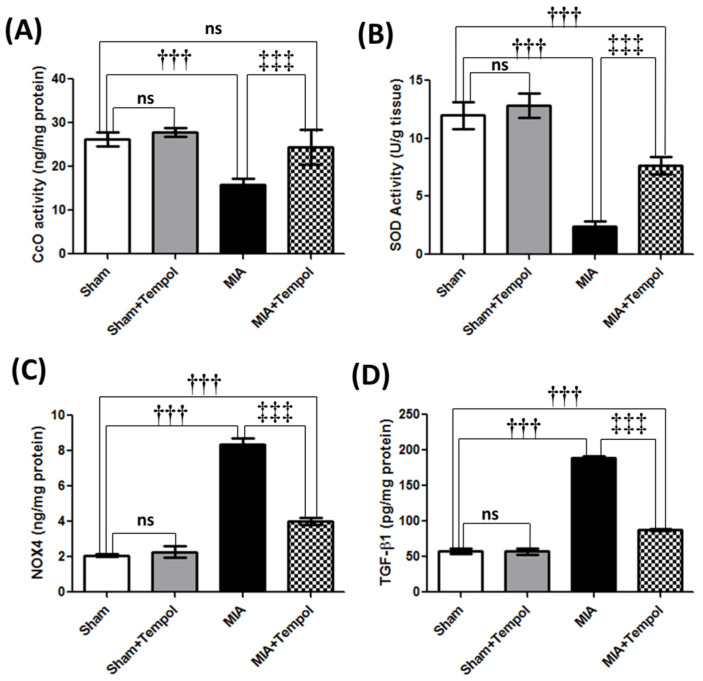
Effect of Tempol on Complex IV oxidase and oxidative stress on osteoarthiritic rats. Rats were subjected to a single intra-articular injection of 3 mg MIA/50 μL saline in their right knees, and then tempol was administered starting from the 7th day of the experiment in a dose of (100 mg/kg/day) by oral gavage for 21 consecutive days. Values of (**A**) Tissue contents of cytochrome c oxidase (CcO) or Complex IV, (**B**) Tissue contents of superoxide dismutase (SOD), (**C**) Tissue contents of NADPH oxidase 4 (NOX4), and (**D**) Tissue contents of transforming growth factor–β1 (TGF-β1). Statistical analysis was carried out using one-way ANOVA followed by Tukey’s multiple comparison test. Results are expressed as mean ± SD (*n* = 6). ^†††^ *p* < 0.001 vs. the sham group, and ^‡‡‡^ *p* < 0.001 vs. MIA group.

MIA disrupted the knee joint activity of CcO (complex IV or the terminal enzyme of the respiratory chain of the mitochondria) by 39.2% (*p* < 0.001) as compared to the sham group (Figure 4A). Moreover, SOD was analyzed calorimetrically and its activity was diminished in the diseased knee joints by MIA by 79.9% (*p* < 0.001) as compared to the sham group (Figure 4B). In contrast, NOX4 which constitutively produces H_2_O_2_ was significantly elevated in the MIA group by 4 folds (*p* < 0.001) compared to the sham group (Figure 4C). Where significant increments in tissue TGF-β1 (3.3 folds, *p* < 0.001) were pronounced in the MIA group when compared to the sham group (Figure 4D). However, tempol post-treatment increased CcO (53.3%) (*p* < 0.001) and SOD (218.7%) (*p* < 0.001) significantly, along with a noticeable decline in NOX4 by 52.0% (*p* < 0.001) and TGF-β1 by (2.1 folds, *p* < 0.001) as relative to the MIA group (Figure 4A–D).

### 2.5. Effect of Tempol on p-SMAD3, and p-p38MAPK Signaling Proteins

**Figure 5:**
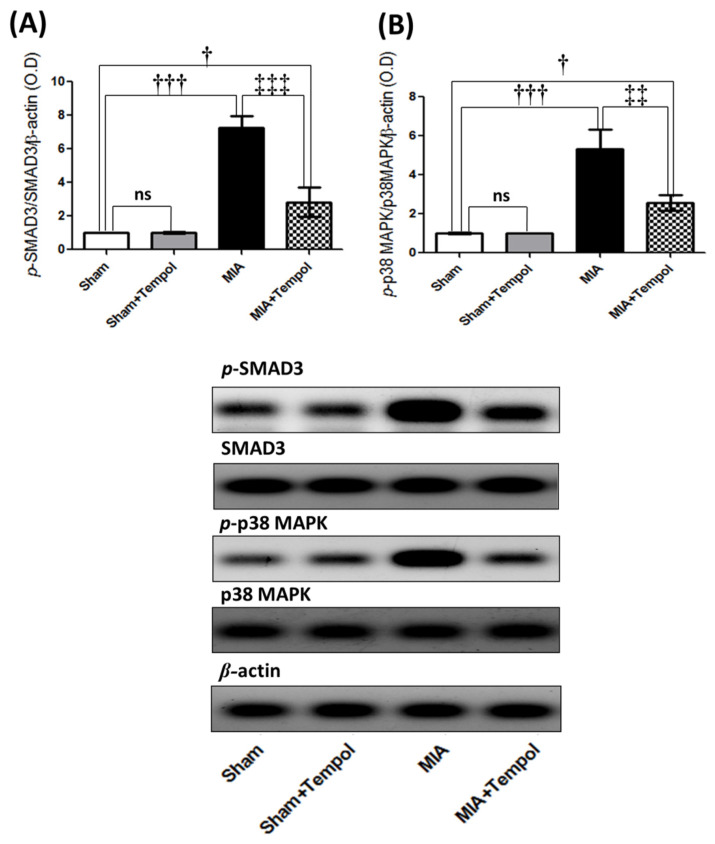
Effect of Tempol intracellular signaling of p-SMAD3 and p-p38MAPK on osteoarthiritic rats. Rats were subjected to a single intra-articular injection of 3 mg MIA/50 μL saline in their right knees, and then tempol was administered starting from the 7th day of the experiment in a dose of (100 mg/kg/day) by oral gavage for 21 consecutive days. Values of (**A**) The protein expression of the phosphorylated small mother against decapentaplegic 3 homologs (p-SMAD3), and (**B**) The protein expression of phosphorylated p38 mitogen-activated protein kinase (p-p38MAPK). The cropped blots of p-SMAD3 and p-p38MAPK were presented relative to that of β-actin, and the uncropped images are available in the Supplementary File. Statistical analysis was carried out using one-way ANOVA followed by Tukey’s multiple comparison test. Results are expressed as mean ± SD (*n* = 3). ^†^ *p* < 0.05 and ^†††^ *p* < 0.001 vs. the sham group, and ^‡‡^ *p* < 0.01 and ^‡‡‡^ *p* < 0.001 vs. MIA group.

Furthermore, MIA insult inappropriately induced marked elevations of the Western blot analyzed phosphorylated proteins; p-SMAD3 (7.2 folds, *p* < 0.001), and p-p38MAPK (5.2 folds, *p* < 0.001) as compared to the sham group. Conversely, tempol post-treatment effectively suppressed the phosphorylated protein levels of p-SMAD3 (60.9%, *p* < 0.001), and p-p38MAPK (51.8%, *p* < 0.01) relative to the MIA-induced OA group (Figure 5).

### 2.6. Effect of Tempol on Inflammatory and Pain Mediators

**Figure 6:**
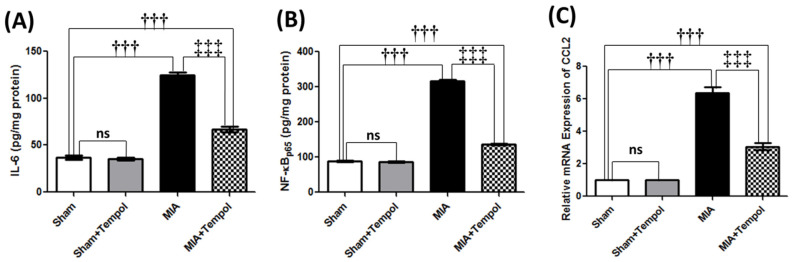
Effect of Tempol on the catabolic inflammatory and pain mediators on osteoarthiritic rats. Rats were subjected to a single intra-articular injection of 3 mg MIA/50 μL saline in their right knees, and then tempol was administered starting from the 7th day of the experiment in a dose of (100 mg/kg/day) by oral gavage for 21 consecutive days. Values of (**A**) Tissue contents of Interleukin-6 (IL-6), (**B**) Tissue contents of nuclear factor-kappa B (NF-κB_p65_), and (**C**) The mRNA expression of chemotactic cytokine ligand 2 (CCL2), are expressed as mean ± SD (*n* = 6). Statistical analysis was carried out using one-way ANOVA followed by Tukey’s multiple comparison test. ^†††^ *p* < 0.001 vs. the sham group, and ^‡‡‡^ *p* < 0.001 vs. MIA group.

The MIA-aggravated inflammatory state was demonstrated by marked escalations of knee joint contents of IL-6 (3.4 folds, *p* < 0.001) and NF-κB (3.6 folds, *p* < 0.001). Moreover, significant rises of the mRNA expression levels of the chemokine CCL2 (6.3 folds, *p* < 0.001) were noticed in the MIA-induced OA group compared to the sham group (Figure 6A–C). In contrast, post-administration of tempol attenuated these elevations, resulting in 46.0% (IL-6, *p* < 0.001), 2.3 folds (NF-κB, *p* < 0.001), and 55.5% (CCL2, *p* < 0.001) lower levels in the tempol-treated group than in the MIA group (Figure 6A–C).

### 2.7. Effect of Tempol on the Radiographic Changes

**Figure 7:**
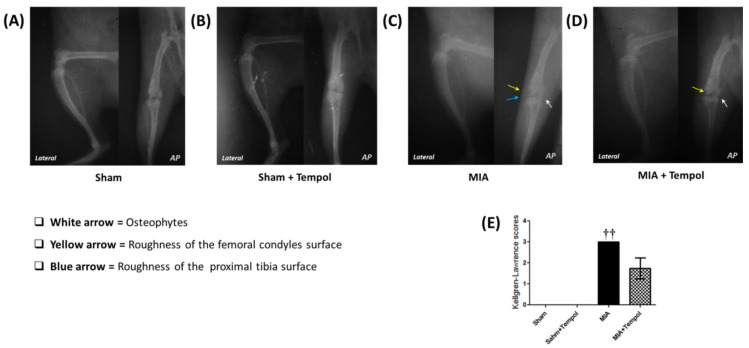
Effect of Tempol on representative X-ray radiographs. Normal radiographs were revealed in the sham group (**A**) and Sham+Tempol group (**B**) along the hip-knee-ankle (HKA) axis. However, the images of the MIA-induced OA group (**C**) showed increased joint opacity, and osteophyte formation (white arrow) associated with rough surfaces of the femoral condyles and proximal tibia (yellow and blue arrow). On the other hand, the X-ray images of the tempol post-treated group (**D**) showed almost normal joint space with little lysis of the femoral articular surface (yellow arrow) and few osteophytes (white arrow). Where AP view is the anterior-posterior view (Flexion angle 10°), and lateral view (Flexion angle 30°). Panel (**E**) represents the means of the Kellgren-Lawrence (K-L) scoring system (Score range: 0–4), where the statistical analysis was carried out using Kruskal-Wallis test followed by Dunn’s post-test. Results are expressed as mean ± SD (*n* = 4). ^††^ *p* < 0.01 vs. the sham group.

The experiment was conducted for X-ray evaluation on day 28. The X-ray images of the right knee joints of the sham groups (Figure 7A,B) showed normal joint structure, normal joint spaces, normal radio-density, and normal surfaces of the femoral condyles and proximal tibia. On the other hand, the osteoarthritic changes of the MIA-induced group (Figure 7C) revealed increased joint opacity corresponding to joint effusion and osteophytes formation; moreover, rough surfaces of the femoral condyles and proximal tibia were also observed corresponding to the articular surfaces lysis. However, the radiographs related to tempol post-treated rats showed almost normal joint space with little lysis of the femoral articular surface, and few osteophytes (Figure 7D). According to Kellgren-Lawrence (K-L) scoring system (Figure 7E), sham groups had the minimum score (grade 0), while the MIA group had the highest score (grade 3). However, the group treated with tempol showed scores not exceeding grade 2.

### 2.8. Effect of Tempol on the Histopathological Alterations

**Figure 8:**
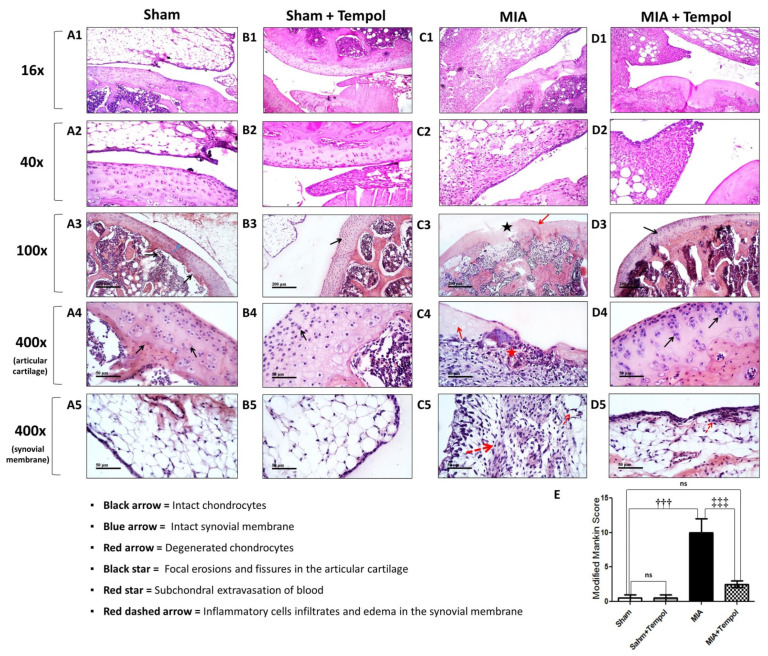
Effect of Tempol on representative H&E photomicrographs. The micrographs of the sham groups revealed normal histological structure of the chondroblasts in the outer surface and inner surface of the articular cartilage and the outer layer of epithelial cell and the inner collagen layer (**A1**,**A2**,**B1**,**B2**) with apparent intact isogenous chondrocytes (black arrow) and regular smooth articular surfaces (**A3**,**B3**). Intact synovial membranes and normal blood vessels are recorded as a blue arrow in the sections of (**A5**,**B5**). The worst distortion is observed in MIA-induced OA sections, where atrophy was detected in the chondroblasts of the outer surface of the articular cartilage and edema, inflammatory cells infiltration with fat in synovial membrane (**C1**,**C2**). severe degenerative and necrotic changes with significant loss of many chondrocytes (red arrow) are accompanied by many focal erosions and fissures of articular cartilage superficial zones (black star) in (**C3**) photomicrograph with occasional subchondral extravasation of blood (red star) in (**C4**). Significant edema of synovial membranes with many inflammatory cell infiltrates (red dashed arrow) recorded in (**C5**). Photomicrographs of the tempol post-treated group (**D1**,**D2**) showed regeneration in the inner surface of the articular surface with absence of the chondroblasts at the outer surface and thickening in the wall of the synovial membrane. In addition, more intact chondrocytes (black arrows) and regular smooth articular surfaces (**D3**,**D4**). Mild inflammatory cell infiltrates (red dashed arrow) were also observed in synovial membranes (**D5**). Panel (**E**) represents the means of the modified Mankin scoring system (Score range: 0–13, from normal cartilage to the maximal score of osteoarthritis), where the statistical analysis was carried out using one-way ANOVA followed by Tukey’s multiple comparison test. Results are expressed as mean ± SD (*n* = 3). ^†††^ *p* < 0.001 vs. the sham group, and ^‡‡‡^ *p* < 0.001 vs. MIA group.

The sham groups showed normal histological structures of articular cartilage layers with apparent intact isogenous chondrocytes. Regular smooth articular surfaces and intact synovial membrane were also observed (Figure 8(A1–5,B1–5)). Chondroblasts atrophy and degenerative changes of the articular cartilaginous surface were detected in MIA-induced OA photomicrographs along with edema, inflammatory cells infiltration with fat in the synovial membrane (Figure 8(C1,C2)). Additionally, severe degenerative and necrotic changes with significant loss of chondrocytes accompanied by focal erosions and fissures of articular cartilage superficial zones (Figure 8(C3)), in addition to occasional subchondral extravasation of blood (Figure 8(C4)), edema with inflammatory cell infiltrations of synovial membranes (Figure 8(C5)). On the other hand, photomicrographs of the tempol post-treated group revealed regeneration in the inner surface of the articular surface with absence of the chondroblasts at the outer surface and thickening in the wall of the synovial membrane (Figure 8(D1,D2)), significant improvements in chondrocytes (Figure 8(D3,D4)), as well as mild inflammatory cell infiltrates in the synovial membrane (Figure 8(D5)). Modified Mankin score was recorded in the histogram panel (Figure 8E), where MIA group attained the maximal mean of the modified Mankin score of (10 ± 1.2, *p* < 0.001) as compared to the sham group. However, the mean of modified Mankin score of tempol post-treated group was found to be (2.5 ± 0.5, *p* < 0.001) relative to the MIA-OA group.

### 2.9. Effect of Tempol on Histochemical Changes

**Figure 9:**
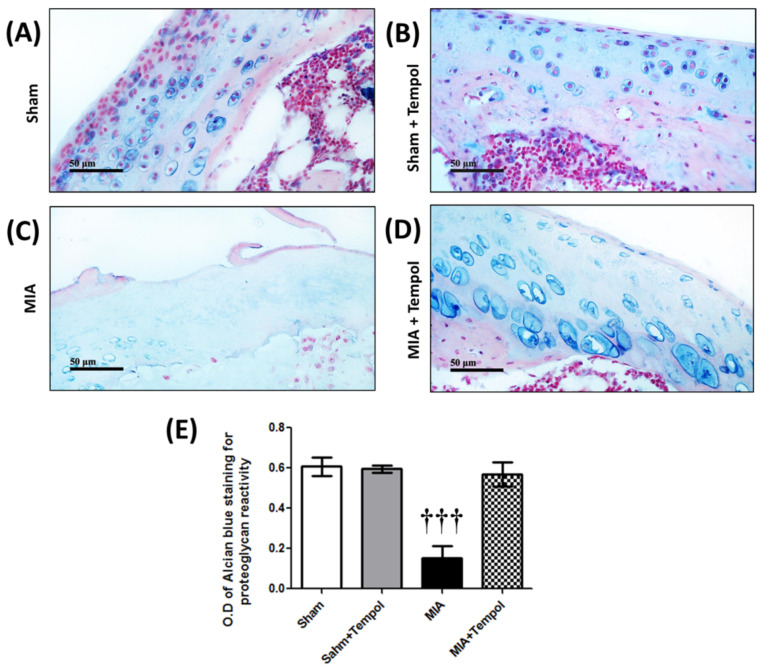
Effect of Tempol on representative alcian blue histochemical photomicrographs. Higher proteoglycans reactivity to alcian blue staining was also observed in (**A**,**B**). The MIA group demonstrated severe loss of proteoglycans reactivity to alcian blue staining all over the articular surfaces (**C**). Post-treatment with tempol (**D**) retained the higher proteoglycan reactivity. Moreover, the histogram panel (**E**) represents the analysis of optical density (O. D) of the alcian blue staining among the studied groups. Where the statistical analysis was carried out using one-way ANOVA followed by Tukey’s multiple comparison test. Results are expressed as mean ± SD (*n* = 3). ^†††^ *p* < 0.001 vs. the sham group.

Well-organized cartilaginous matrixes were recorded with strong high density and reactivity of proteoglycans to alcian blue stain (Figure 9A,B). Significant loss of proteoglycans reactivity to alcian blue was recorded all over articular surfaces in the MIA group (Figure 9C). Moreover, post-treatment with tempol was able to retain the higher reactivity of proteoglycan (Figure 9D).

## 3. Discussion

The present study demonstrates the effective role of tempol as a membrane-permeable radical scavenger in attenuating the related physical, structural, functional, and biochemical deleterious alterations of the knee osteoarthritis rodent model. Accordingly, OA was induced by a single unilateral intra-articular injection of monosodium iodoacetate (MIA) in the rat’s right knee joint. In the current investigation, the degenerative effects of MIA on cartilage are demonstrated biochemically by predominant elevations of MMP-13, fibulin-3 expression, and bone ALP activity in the MIA group, our results are consistent with previous studies [24,25]. Fibulin-3 is a member of the ECM proteins that provides organization and stabilization to ECM structure [26]. Bone ALP is expressed in injured cartilage tissues on the cell surface and within matrix vesicles [27]. In addition, these findings are accompanied by MIA-related radiological and histological deteriorations which is similar to other studies [5,10,25].

Contrariwise, the post-treatment of tempol repressed all MIA-induced elevations of knee joint degradation-related biomarkers. Moreover, tempol showed few osteophytes in the related radiographs, and also retained the higher proteoglycan reactivity in response to alcian blue staining. Thus, these outcomes suggest the potential role of tempol in reversing the MIA-destructive effect on the cartilage and further highlight the anti-osteoarthritic efficacy of tempol.

It was demonstrated that MIA disrupts glycolysis and aerobic cellular respiration via inhibition of glyceraldehyde-3-phosphate dehydrogenase (GAPDH) [5,28]. As a consequence, MIA instigated oxidative phosphorylation disruption, ROS elevation, the release of cytochrome c from the mitochondrion, activation of caspase-3, and finally provoking chondrocytes apoptosis in rats [29]. Hence in our study, cytochrome c oxidase (CcO, complex IV) activity was diminished in the MIA group. CcO is the terminal electron acceptor in the electron transport chain (ETC) and the regulation site for mitochondrial oxidative phosphorylation sequel [30]. Thus, our findings indicated that oxidative phosphorylation and mitochondrial energy homeostasis were disrupted following MIA insult. In the current investigation, MIA insult accompanied the perturbed oxidative phosphorylation by oxidative stress status of lower SOD activity and elevated NOX4 contents, these results are consistent with previous studies [31,32]. Basically, intracellular ROS such as superoxide anion (O_2_^−^) and H_2_O_2_ are derived from mitochondria and membrane NADPH oxidase (NOX) systems [33]. The O_2_^−^ reacts with biomolecules to cause molecular damage or turn spontaneously into H_2_O_2_ or diminished by the SOD antioxidant activity [34]. Inappropriately, SODs are expressed at lower levels in OA cartilage [35]. However, it has been reported that NOX4 is the predominant isoform active in chondrocytes of OA cartilage [36]. NOX4 constitutively produces H_2_O_2_ [37]. NOX4-derived ROS generation triggers many transcriptional events responsible for inflammatory cytokines production, lipid peroxidation, mitochondrial DNA damage, MMPs production, and cartilage breakdown exacerbation [13,31]. Eventually, these events lead to chondrocyte cell death, osteophyte formation, synovial fibrosis, and joint pain in animal models [38,39].

On the contrary, CcO was replenished in the tempol-treated rats. Likewise, a previous study has demonstrated that tempol maintained the mitochondrial respiratory function in cisplatin-induced nephrotoxicity in mice [40]. In addition, tempol post-treatment mitigated MIA-induced oxidative stress via replenishing SOD and diminishing NOX4 activity. These findings reasonably rely on the anti-oxidant efficacy of tempol. Interestingly, these results can collectively support the linkage between the anti-oxidant effect of tempol and its ability to maintain mitochondrial function dynamics and cellular redox balance which primarily emphasizes the potential role of tempol in alleviating cartilage degeneration.

It is believed that TGF-β has a central role in the pathogenesis of OA, it is implicated in increasing MMP-13 expression [39]. When active TGF β is released from the ECM, it binds to TGF β receptor and directly activates receptor-regulated SMAD3 [39] which subsequently activates the target transcription of NADPH oxidase 4 (NOX4) [18] and MAPK signaling leads to osteophyte formation, synovial fibrosis, and joint pain in animal models as declared previously [38,39]. MAPKs are activated in joint disorders and that is associated with MIA-induced pain as advocated by previous studies [11,41]. Hereby, our findings demonstrate that MIA injection resulted in elevations of anabolic growth factor TGF-β1, p-SMAD3, and p-p38MAPK. In contrast, tempol suppressed all these elevations. Relying on these findings, the anti-oxidant mechanism of tempol is not related only to its intracellular redox cycling negotiation, but it is also related to the ability of tempol to suppress the TGF-β1/SMAD3/NOX4 pathway, which further diminishes NOX4-derived ROS generation sequels.

In the present study, MIA prompted an inflammatory response that was noticeably presented by increasing knee joint diameter and swelling especially during the first 10 days of the experimental period, and then subsided gradually, these outcomes were consistent with previous studies [24,31]. The MIA-related histological micrographs were accompanied by edema and inflammatory cells infiltration of the synovial membrane. Likewise, the MIA-induced biochemical alterations showed significant elevations of the pro-inflammatory mediators including tissue contents of IL-6 and NF-κB_p65_, and CCL2 expressions in OA rats, these outcomes were previously mentioned [5,42,43]. It has been noted that the abnormally generated ROS exacerbates the inflammatory process in OA and aggravates ECM and joint degradation [44]. Inflammation is the most significant cause of OA-related structural changes [45]. During the progress of OA, chondrocytes and synoviocytes stimulate the production of inflammatory mediators [46]. CCL2 or called monocyte chemoattractant protein-1 (MCP-1), is well known to facilitate the migration and infiltration of monocytes and macrophages [47]. The stress kinase, p38MAPK is activated in response to the inflammatory cytokines, oxidative stress, and growth factors activation [19]. Activation of p38MAPK triggers NF-κB signaling activity and its sequels [48]. The abnormal activation of NF-κB in OA coordinates a complex-multilayered signaling network leading to the up-regulation of catabolic factors include cytokines, chemokines, prostaglandin E2, and MMPs, which eventually lead to cartilage damage and osteophytes formation [49].

On the other hand, the post-treatment of tempol subsided all the forgoing MIA-induced inflammatory mediators’ release. Additionally, the tempol-treated rats exhibited marked joint swelling relief starting from the 4th day of treatment, and histologically showed mild inflammatory cell infiltrates in the synovial membrane. Previous studies have demonstrated the anti-inflammatory effect of tempol, for example, tempol enhanced the periodontitis in a rodent model [50]. Additionally, tempol delayed the altered investigations of the collagen-induced arthritis rat model [23]. Accordingly, our findings indicate the potential anti-inflammatory effect of tempol on MIA-induced knee OA.

In this knee OA model, the accelerating rotarod performance test was used for the functional assessment of MIA-induced pain and motor imbalance. The osteoarthritic rats displayed poor performance in the rotarod which was presented by gradual reduction of the latency time to fall throughout the experimental schedule, these findings are in harmony with previous studies [25,42,51]. The production of chemokines, cytokines, and proteases sensitizes primary sensory neuron (PSN) afferents [11]. CCL2 stimulates direct excitation on nociceptive neurons and microglial activation which further triggers persistent hyperalgesia [47]. NF-κB/IL-6 signaling activation was documented to be interconnected with chronic pain sensitivities and cartilage damage in OA [52]. Additionally, ROS also participates in the sensitization of the dorsal horn neurons, provoking the central sensitization through glial activation [53].

Tempol-treated rats exhibited improved motor performance noticed by the significant increase of the latency time to fall on day 21 and day 28, whereas earlier studies demonstrated the analgesic effects of tempol in attenuating neuropathic pain of spinal nerve ligation [54] or preventing the pain sensitization after capsaicin injection into the foot [55]. Thus, our attained findings recommend the potential analgesic activity of tempol inferred to its anti-inflammatory effects in the MIA-induced OA model in rats. Collectively, these outcomes suggest the interconnection between the anti-oxidant effects of tempol and its ability to subside the catabolic inflammatory cascades including inflammatory cell infiltration, angiogenesis, and pain which further underline the anti-osteoarthritic role of tempol in the MIA-OA model.

Four limitations of this study are identified. First, chemical chondrotoxic agents such as MIA are reported to have unique pathophysiology that has no association with post-traumatic OA. Therefore, this method may be useful for determining whether osteoarthritic changes originate from cartilage or subchondral bone alterations and it is mainly used to observe and evaluate pain behavior and to assess the efficacy of therapeutic interventions to reduce inflammation and pain. Second, this study did not compare the tested tempol with appropriate pharmacological control. Third, this study needs a further demonstration of the autophagy picture by evaluating the lysosomal formation and/or fusion with the autophagosomes. Another limitation of this study is the unavailability to investigate the in vitro signal transductions in chondrocytes.

In summary, the potential anti-oxidant and anti-inflammatory mechanisms of tempol on MIA-induced osteoarthritis in rats can be collectively demonstrated through its ability to provoke collateral suppression of the catabolic signaling cascades including TGF-β1/SMAD3/NOX4, and NOX4/ROS/p38MAPK/NF-κB, which in turn results in decreasing MMPs production, reducing catabolic inflammatory sequels, limiting structural alterations, and reducing pain.

## 4. Materials and Methods

### 4.1. Ethical Statement

All procedures for handling, use, and euthanasia of animals were reviewed and approved by the Ethical Committee for Animal Experimentation of the Faculty of Pharmacy, Cairo University, with the permit number PT 2389, and were performed following the guides for the Care and Use of Laboratory Animals published by the US National Institutes of Health (8th edition, NIH Publication, 2011).

### 4.2. Experimental Animals

Forty adult male Wistar albino rats (180–200 g; 8–10 weeks old) were obtained from the National Research Centre’s Animal House Colony (NRC, Giza, Egypt). The rats were randomly divided into four groups (5/cage) in a room with a controlled temperature (22 °C ± 1) on a 12/12 light-dark cycle. All rats were fed standard rat chow and had access to water. The animals were housed in the same environment for 2 weeks prior to experiment. Adequate non-pharmacological measures were taken to minimize animal pain or discomfort according to the National Centre for the Replacement, Refinement and Reduction of Animals in Research (NC3Rs) strategy and Animal Research: Reporting of In vivo Experiments (ARRIVE) guidelines.

### 4.3. Induction of Osteoarthritis

Single unilateral intra-articular injection of monosodium iodoacetate (MIA) was used to induce knee osteoarthritis in rats (Sigma-Aldrich Chemical Co., Lot# SLBZ7569, St. Louis, MO, USA). On day zero, rats were anesthetized with 4% isoflurane inhalation. Three mg of MIA dissolved was dissolved in 50 μL of sterile saline and injected through the patellar ligament into the joint space of the right knee using a sterile 100-U insulin syringe [10,31,32,45,56,57]. The sham groups were injected with 50 μL physiologic saline were injected in the right knees of the sham groups instead of MIA [42].

### 4.4. Drugs

Tempol (Sigma-Aldrich Chemical Company, St. Louis, MO, USA) was prepared and dissolved daily in distilled water in a concentration of 0.4 g/20 mL. The choice of tempol dose was on a pilot trial (Supplementary Materials) guided by previously published studies to figure out the appropriate method of administration [40,58,59,60], and the applicable duration of treatment [31,42,44,56,61].

In this study, we did not compare with a pharmacological control since the existing approved pharmacological treatments for OA are limited for the relief of OA symptoms (e.g., NSAIDs and corticosteroids), and there is a lack of licensed disease-modifying osteoarthritis drugs (DMOADs) that could slow the narrowing of joint space and provide symptomatic relief.

### 4.5. Experimental Design

**Figure 10:**
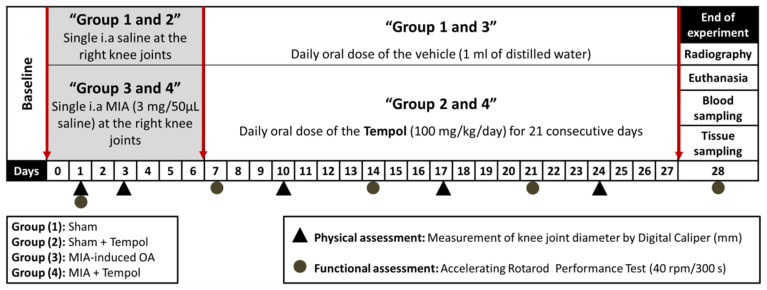
Schematic diagram of the experimental protocol.

The experimental protocol is summarized in (Figure 10). Forty adult male rats were randomly allocated into four groups (*n* = 10/group). In the first sham group, the animals were subjected to a single intra-articular injection of 50 μL normal saline in their right knee joints on day 0 and then administered a daily oral dose of the vehicle (1 mL of distilled water), one week after saline injection for 21 days. Rats in the second group (sham + Tempol) received an intra-articular injection of normal saline in their right knee joints, followed by oral gavage of tempol (100 mg/kg/day) for 21 days one week after saline injection. On day 0, osteoarthritis was induced by single unilateral intra-articular injection of MIA in a dose of 3 mg dissolved in 50 μL saline in group 3 (MIA-induced OA) and group 4 (MIA + Tempol). Then the third group administered vehicle (1 mL of distilled water) for 21 days starting from the 7th day of the experiment. While, in the fourth MIA + Tempol group, the osteoarthritic rats were administered tempol starting from the 7th day of the experiment post-MIA in a dose of (100 mg/kg/day) by oral gavage for 21 consecutive days.

### 4.6. Assessment of Knee Joint Edema

The diameter (in mm) of the right knee of each rat in the studied groups (*n* = 8/group) was measured weekly using a calibrated digital caliper (SL-1112, INSIZE Co., Loganville, GA, USA), at the corresponding days 0, 1, 3, 10, 17, and 24 [24].

### 4.7. Evaluation of Motor Performance Using Accelerating Rotarod Test

The accelerating rotarod apparatus (Model 7750, Ugo Basile, Italy) was used to assess motor stability, coordination, and pain. Each rat was trained for three days (one session/day) just before the experimentation and the non-responsive rats during training were excluded from rotarod testing. Then, rats were randomly placed on the rotating rod and forced to walk at increasing speeds for 5 min (300 s) [25]. Throughout the experiment, rats were tested for persistence on the accelerating rotarod on study days 1, 7, 14, 21, and 28. The duration of each rat’s grip on the rod was recorded as latency time to fall in seconds (*n* = 8/group) [51,62].

Assessment of knee joint edema by digital caliper and evaluation of motor performance by accelerating rotarod were performed on different experiment days to avoid conflicting outcomes from the two tests together and also to avoid an extra effort or extra stress on rats which could be counterproductive.

### 4.8. Radiographical Examination

On day 28, rats were anesthetized by ketamine/xylazine (100/10 mg/kg, i.p.). Afterward, the rat’s right knee was flexed in the anterior to posterior position (AP view, angle 10°), and lateral position (angle 30) [63]. The X-ray films were captured by 50 mA mobile X-ray camera apparatus (Model F50-100II 50mA, Perlong Medical Equipment Co., Nanjing, China). Two investigators blindly examined all X-ray images. The severity of joint lesions (*n* = 4/group) was evaluated according to the Kellgren-Lawrence (K-L) scoring system. The score ranged from 0 to 4, resulting in five grades: none, doubtful, minimal, moderate, and severe [64].

### 4.9. Tissue Sampling

On day 28, animals were euthanized with pentobarbital sodium (200 mg/kg, i.p.). The right knee joints were separated into two sets. The first set of tissue samples (*n* = 6) was used for the colorimetric assay of SOD activity according to the method of Nishikimi et al. [65], ELISA assays, real-time polymerase chain reaction (RT-PCR) analysis, and Western blot analysis (*n* = 3). The second tissue set (*n* = 3) was fixed in 10% formalin for histopathological evaluation.

### 4.10. Biochemical ELISA Measurements

Knee joint tissues were minced, placed in a round bottom microtube, snap-frozen in liquid nitrogen, and homogenized with an electric homogenizer in five volumes of extraction buffer (100 mM Tris, pH 7.4, 150 mM NaCl, 1 mM EGTA, 1 mM EDTA, 1% Triton X-100, and 0.5% sodium deoxycholate). The tissue lysates were placed on a shaker at 4 °C for 1 h and centrifuged at 10,000× *g* for 5 min. The supernatants were stored at −80 °C for further analysis of Bone alkaline phosphatase (ALP; CSB-E11865r, Cusabio Biotech Co., Wuhan, China), cytochrome c oxidase (complex IV) (CcO; CSB-E14281r, Cusabio Biotech Co., Wuhan, China), interleukin-6 (IL-6; R6000B, R&D Systems, Shanghai, China), matrix metalloproteinase-13 (MMP-13; CSB-E07412r, Cusabio Biotech Co., Wuhan, China), NADPH oxidase 4 (NOX4; LS-F5720, LSBio, Seattle, WA, USA), nuclear factor-kappa B-p65 subunit (NF-κB_p65_; MBS722386, MyBioSource, San Diego, CA, USA), and transforming growth factor-β1 (TGF-β1; MBS175833, MyBioSource, San Diego, CA, USA) using rat specific ELISA kits according to the manufacturer’s instructions.

### 4.11. Quantitative Real-Time PCR (qRT-PCR) Measurements for Gene Expression

The messenger RNA (mRNA) expression levels of chemokine (C-C motif) ligand 2 (CCL2), and fibulin-3 were analyzed by quantitative real-time PCR. About 30 mg of knee joint tissue was homogenized in RNA lysis solution supplied by RNeasy mini kit (QIAGEN, Maryland, CA, USA) and centrifuged at 10,000× *g* for 10 min, the supernatant was used for RNA extraction according to the manufacturer’s protocol. The concentrations of the isolated RNA were obtained using ultraviolet spectrophotometry, and RNA purity was assessed based on the A260/A280 absorption ratio. Then, RNA was reverse-transcribed into cDNA as described in the manufacturer’s using High Capacity cDNA reverse transcription kit (Applied Biosystems, Foster City, CA, USA). Real-time PCR was performed with a PCR mixture containing 1 µmol/L of each primer and SYBR Green Master Mix Applied Biosystems, Foster City, CA, USA) using StepOne™ PCR system (version 3.1, Applied Biosystems, Foster City, CA, USA). The sequences of the primers used are listed in (Table 1). All primer sets had a calculated annealing temperature of 60 °C. Amplification conditions were: 95 °C for 10 min, and then 40 cycles of denaturation for 15 s and annealing/extension at 60 °C for 10 min. All values were normalized to the β-actin which was used as the endogenous control (reference gene). The relative expression of the target genes was obtained using the comparative threshold cycle CT (ΔΔCT) method. The relative expression was calculated from the 2^−ΔΔCT^ formula [66].

### 4.12. Western Blot Analysis

Frozen knee joints were weighed and broken into pieces on dry ice. Then were homogenized in Radio Immunoprecipitation Assay (RIPA) lysis buffer (50 mM Tris-HCl, 150 mM NaCl, 1% Triton X-100, and 1% deoxycholate) supplemented with 15 mM sodium fluoride, 1 mM sodium vanadate, 2 mM sodium pyrophosphate, 1 mM sodium glycerophosphate, 2 mM imidazole, 100 mg/mL phenylmethylsulfonyl fluoride and proteinase inhibitor cocktail (Thermo Fisher Scientific Inc., Waltham, MA, USA), and sonicated in the lysis buffer, cleared by centrifugation at 12,000× *g* for 10 min at 4 °C. The protein was quantified using Bradford Protein Assay Kit (Bio Basic Inc. Markham, ON, Canada) [67] and kept at −80 °C until use. Briefly, 50 µg total protein samples were resolved on sodium dodecyl sulfate (SDS)-polyacrylamide gel electrophoresis (PAGE) gels which were prepared using TGX Stain-Free™ FastCast™ Acrylamide Kit (Bio-Rad Laboratories, Inc., Hercules, CA, USA), and then transferred to polyvinylidene difluoride (PVDF) membranes (Sigma-Aldrich Chemical Co., St. Louis, MO, USA), using a semi-dry transfer apparatus Bio-Rad Trans-Blot Turbo Apparatus (Bio-Rad Laboratories Inc., Hercules, CA, USA). The membranes were blocked with 3% bovine serum albumin (BSA) in Tris-buffered saline with Tween-20 (TBST) at 4 °C for 1 hr. The membranes were then incubated overnight on a roller shaker at 4 °C with the following primary rabbit/IgG polyclonal antibodies (Thermo Fisher Scientific Inc., Waltham, MA, USA): anti-small mother against decapentaplegic 3 homologs (SMAD3) (1:1000; PA5-17378), anti-phospho-(p)-SMAD3 (1:1000, Cat# PA5-104942), anti-p38 mitogen-activated protein kinase (p38MAPK) (1:200, Cat# OPA1-10080), and anti-phospho-(p)-p38 MAPK (1:1000, Cat# 44-684G). After washing in TBST, the membranes were probed with HRP-conjugated goat anti-rabbit IgG secondary antibody (1:1000, Cat# HAF008, Novus Biologicals, -Centennial, CO, USA) at 4 °C for 1 h. Finally, the protein bands were developed using a ClarityTM Western ECL chemiluminescent substrate (Cat# 170-5060, Bio-Rad Laboratories Inc., Hercules, CA, USA). The optical densities (O.D) of the expressed proteins were analyzed by ChemiDoc™ MP Imaging System using Image Lab™ Software (version 5.1, Bio-Rad Laboratories Inc., Hercules, CA, USA). The O.D of the subsequently mentioned results was normalized to β-actin.

### 4.13. Histopathological Examinations

Autopsy samples were taken from the rats’ right knee joint of different groups (*n* = 3/group) and fixed in 10% neutral buffered formalin for 48 hr. Followed by decalcification using Cal-Ex™ II Fixative/Decalcifier (Fisher Chemical™ Scientific, Waltham, MA, USA) for 20 days. Joints were trimmed and sectioned at the mid-sagittal point then serial step sections were obtained and examined at different levels. Subsequently, samples were processed using serial dilutions of ethanol and cleared in xylene followed by infiltration and embedding in paraplast tissue embedding media. Tissue sections (5 μm thickness) were made by rotatory microtome and mounted on glass slides for hematoxylin and eosin (H&E) staining for general histological examination of joint samples, and also for histochemical assessment by alcian blue stain (AB; pH 2.5) for assessment of cartilage extracellular matrix and proteoglycans reactivity. Nuclear fast red was used as a standard counterstain for alcian blue staining. All guidelines for sample fixation, processing, and staining were done according to Culling et al. [68]. Morphological assessment of tibiofemoral articular cartilage was conducted according to the modified Mankin scoring system [69,70] with 0 indicating normal cartilage and 13 indicating the maximal score of osteoarthritis. In addition, six random non-overlapping fields from alcian blue stained articular cartilage of each sample were quantitatively assessed for their absorbance as mean optical density (O.D.) between different groups. All data were obtained by the Leica image analyzer computer system attached to a full HD microscopic imaging system (Leica Microsystems GmbH, Wetzlar, Germany).

### 4.14. Statistical Analysis

Statistical comparisons between different means of parametric data (*n* = 6 for biochemical analysis by ELISA and qRT-PCR or *n* = 3 for Western blot analysis or *n* = 3 for histological analysis) were carried out using one-way analysis of variance (ANOVA) followed by Tukey’s multiple comparison test. Furthermore, knee joint diameter and rotarod latency time were tabulated and analyzed statistically using repeated measures ANOVA test for factors time and group, followed by the Bonferroni post-hoc test. the radiologic scores were analyzed using Kruskal-Wallis test followed by Dunn’s post-test (*n* = 3). Statistical analysis was performed using GraphPad Prism (version 5.0, GraphPad Software, Inc., San Diego, CA, USA). All data points are presented as the means ± SD. A difference was considered to be statistically significant at *p* < 0.05.

## 5. Conclusions

The potential anti-oxidant and anti-inflammatory mechanisms of tempol on MIA-induced osteoarthritis in rats can be collectively demonstrated through its ability to provoke collateral suppression of the catabolic signaling cascades including TGF-β1/SMAD3/NOX4, and NOX4/ROS/p38MAPK/NF-κB, which in turn results in decreasing MMPs production, reducing catabolic inflammatory sequels, limiting structural alterations, and reducing pain.

## Figures and Tables

**Table 1:** The primer sequences for analyzing mRNA expression of CCL2 and fibulin-3. F: forward primer, mRNA: messenger RNA and R: reverse primer.

mRNA Species	Primer Sequence (5′‒3′)
chemokine (C-C motif) ligand 2 (CCL2)	F: AGCCAACTCTCACTGAAGC R: GTGAATTGAGTAGCAGCAGGT
Fibulin-3 (fib-3)	F: TGTGACCCAGGATATGAACTTGAG R: AGCCCCCTTGTAGATTGTAGCA
β-actin	F: CCAACCGCGAGAAGATGA R: CCAGAGGCGTACAGGGATAG

## Data Availability

Datasets collected or analyzed during the current study are available from the corresponding author on request.

## Supplementary Materials

The following are available online. Table S1: The serum levels of crosslinked C-telopeptides of type II collagen (CTX-II) and Table S2: The serum levels of Interleukin-1β (IL-1β).

## Sample Availability

Samples of the compounds are available from the authors.
