# Supplementary material for: In Vivo Investigation of the Ameliorating Effect of Tempol against MIA-Induced Knee Osteoarthritis in Rats: Involvement of TGF-β1/SMAD3/NOX4 Cue
Source: Molecules. 2021 Nov 19;26(22):6993. doi: 10.3390/molecules26226993 (PMC8618489; doi:10.3390/molecules26226993)
Supplement: Supplementary file 1 [file molecules-26-06993-s001.zip › molecules-1451169-supplementary.pdf]

Pilot study

"Effect of tempol (25, 50, an100 mg) on MIA-induced OA in rats"

The pilot study assessed the effect of 3 different doses of tempol (25, 50, and 100 mg/kg) using X-ray investigation and serum CTX-II and IL-1β.

Table S1. The serum levels of crosslinked C-telopeptides of type II collagen (CTX-II) is expressed as mean ± SEM (n=3-4). Statistical analysis was carried out using one-way ANOVA followed by Tukey's multiple comparison test. P<0.05. Significance from sham group (*), Significance from MIA-induced OA group (#), Significance from TEMPOL 25 (@).

Group	CTX-II
**SHAM**	0.70±0.15
**MIA**	8.85±0.96 *
**TEMPOL (25 mg/kg)**	6.23±0.49 *
**TEMPOL (50 mg/kg)**	5.38±0.53 *
**TEMPOL (100 mg/kg)**	2.5±0.26 #@

Table S2: The serum levels of Interleukin-1β (IL-1β) is expressed as mean ± SEM (n=3-4). Statistical analysis was carried out using one-way ANOVA followed by Tukey's multiple comparison test. P<0.05. Significance from sham group (*), Significance from MIA-induced OA group (#), Significance from TEMPOL 25 (@), Significance from TEMPOL 50 ($).

Group	IL-1β
**SHAM**	41.23±2.90
**MIA**	119.7±3.30 *
**TEMPOL (25 mg/kg)**	94.23±2.5 *#
**TEMPOL (50 mg/kg)**	86.45±4.00 *#
**TEMPOL (100 mg/kg)**	58.20±6.03 #@$
